# Impact of cerebral small vessel disease burden and systemic clinical phenotypes on short-term neurological outcomes after acute ischemic stroke

**DOI:** 10.3389/fneur.2026.1799749

**Published:** 2026-06-04

**Authors:** Lifang Ma, Fangtong Liu, Jing Deng, Fangyuan Cui, Jing Bai, Bin Ma, Lu Tang, Xiao Han, Li Zhou, Ying Gao, Yan Li

**Affiliations:** Dongzhimen Hospital, Beijing University of Chinese Medicine, Beijing, China

**Keywords:** acute ischemic stroke, cerebral small vessel disease, MRI, neurological outcome, risk stratification

## Abstract

**Introduction:**

Cerebral small vessel disease (CSVD) is increasingly recognized as an important determinant of outcome after acute ischemic stroke (AIS). However, the contribution of global CSVD burden, individual imaging markers, and systemic clinical phenotypes to early neurological recovery remains incompletely understood.

**Methods:**

We conducted a retrospective cohort study of consecutive AIS patients admitted to Dongzhimen Hospital, Beijing University of Chinese Medicine between January and December 2024. CSVD markers were assessed using MRI according to STRIVE criteria, and a total CSVD burden score (0–4) was calculated. Systemic clinical phenotypes were defined based on standardized admission assessments. The primary outcome was unfavorable neurological status at discharge (NIHSS >8). Multivariable logistic regression with collinearity diagnostics was performed. Distributions of NIHSS scores across CSVD features were visualized using violin plots.

**Results:**

A total of 474 patients were included (median age 67 years; 71% male), of whom 31 (6.5%) had unfavorable outcomes. Higher total CSVD burden (adjusted OR 1.57, 95% CI 1.13–2.17), hyperhomocysteinemia (adjusted OR 2.76, 95% CI 1.21–6.31), and a Traditional Chinese Medicine (TCM)-defined Phlegm-Heat Fu-Excess phenotype (adjusted OR 5.28, 95% CI 1.85–15.04) were independently associated with unfavorable outcomes. Among individual CSVD markers, lacunar infarction showed the strongest association. White matter hyperintensities, cerebral atrophy, and basal ganglia enlarged perivascular spaces were associated with higher discharge NIHSS scores in exploratory analyses. Given the limited number of outcome events, the stability of the regression model may be limited, and these findings should be interpreted with caution.

**Discussion:**

Global CSVD burden and selected systemic clinical phenotypes are associated with poor short-term neurological outcomes after AIS. These findings should be considered exploratory and hypothesis-generating, and may provide supplementary information for early risk stratification. Further validation in larger, prospective multicenter studies is required.

## 1 Introduction

Acute ischemic stroke (AIS) remains a leading cause of mortality and long-term disability worldwide. Although advances in reperfusion therapy and stroke unit care have improved outcomes, a substantial proportion of patients experience poor neurological recovery at discharge, limiting rehabilitation potential, and functional independence (1). Early identification of patients at high risk for unfavorable outcomes remains a major clinical challenge.

Cerebral small vessel disease (CSVD) represents a chronic and progressive microangiopathy that accounts for approximately one-quarter of all ischemic strokes (2). Silent CSVD is extremely common as we age, affecting most people above 80 (3). Comorbid CSVD is highly prevalent in the AIS population, and accumulating evidence links it to an increased risk of both incident and recurrent stroke (4, 5). This creates a common clinical scenario: patients suffering from an acute vessel occlusion often concurrently bear a chronic, insidious burden of microvascular injury. The influence of this underlying CSVD burden—a potential reflection of cerebral frailty and may indicate impaired vascular reserve—on short-term neurological outcomes remains insufficiently investigated. MRI manifestations of CSVD—including white matter hyperintensities, lacunar infarcts, cerebral microbleeds, enlarged perivascular spaces, and cerebral atrophy—could be associated with cumulative microvascular injury and may suggest reduced cerebral resilience. While CSVD is well established as a predictor of cognitive impairment, recurrent stroke, and long-term disability, its influence on early neurological recovery following AIS has been less extensively studied.

In addition to neuroimaging markers, systemic clinical conditions may also influence stroke outcomes. Previous studies have suggested potential links between certain traditional Chinese medicine (TCM)-based systemic phenotypes and inflammatory or metabolic burden, although direct measurements of corresponding biomarkers were not available in the present study (6–8). However, the combined contribution of CSVD burden and systemic clinical phenotypes to short-term neurological outcomes has not been comprehensively evaluated.

Therefore, this study aimed to investigate the association between total CSVD burden, individual CSVD imaging markers, and systemic clinical phenotypes with neurological status at discharge in patients with AIS. We hypothesized that higher CSVD burden and adverse systemic clinical phenotypes would be associated with unfavorable early neurological outcomes.

## 2 Materials and methods

### 2.1 Study design and participants

This retrospective cohort study included consecutive patients admitted with a primary diagnosis of AIS to Dongzhimen Hospital, Beijing University of Chinese Medicine, between January and December 2024.

Inclusion criteria were: age ≥18 years; symptom onset within 7 days prior to hospital admission; availability of brain MRI allowing full assessment of CSVD markers; documented NIHSS score at discharge, which was assessed at a mean of 14 ± 3 days after admission.

Patients who underwent thrombolysis or endovascular therapy were excluded to reduce heterogeneity and minimize the confounding effects of acute reperfusion treatments on early neurological outcomes. However, this exclusion may introduce selection bias and limit the generalizability of the findings.

### 2.2 Data collection

Baseline demographic characteristics, medical history (including hypertension, diabetes, hyperhomocysteinemia, etc.), and laboratory parameters collected within 72 h of admission were retrieved from electronic medical records. Laboratory tests included routine blood counts, biochemistry, lipid profile, glycated hemoglobin (HbA1c), homocysteine (Hcy), and coagulation parameters.

MRI markers of CSVD were evaluated according to STRIVE recommendations, including: white matter hyperintensities (WMH, rated using the Fazekas scale), lacunar infarcts (LI), cerebral microbleeds (CMBs), enlarged perivascular spaces (EPVS) in basal ganglia and centrum semiovale (graded using a semi-quantitative scale), and global cerebral atrophy (9). Total CSVD burden was calculated using a validated 0–4 scale: One point was awarded for each of the following four features: (1) presence of one or more lacunes; (2) presence of one or more cerebral microbleeds; (3) moderate-to-severe enlarged perivascular spaces (grade 2–4) in the basal ganglia; and (4) moderate-to-severe white matter hyperintensities, defined as deep white matter hyperintensities with a Fazekas score of 2 or 3, or periventricular hyperintensities with a Fazekas score of 3 (10).

Lacunes were defined as round or ovoid, subcortical, fluid-filled cavities (3–15 mm in diameter) with signal characteristics similar to cerebrospinal fluid on all MRI sequences, in accordance with the STRIVE criteria. To avoid confounding by acute infarction, acute lacunar infarcts were excluded based on diffusion-weighted imaging (DWI) and corresponding FLAIR hyperintensity. Only chronic lacunes were included as markers of CSVD in this study.

All subjects were scanned using a 3.0 T MRI system (Siemens, Prisma, Germany). The imaging protocol included high-resolution T1-weighted imaging, T2-weighted imaging, fluid-attenuated inversion recovery (FLAIR), susceptibility-weighted imaging (SWI), and diffusion-weighted imaging (DWI) sequences. Imaging was performed using standardized clinical protocols to ensure consistency and reproducibility.

All MRI scans were independently evaluated by two senior neurologists who were blinded to clinical and outcome data.

In cases of disagreement between the two raters, a consensus was reached through discussion. If consensus could not be achieved, a third senior neuroradiologist was consulted for final adjudication.

Systemic clinical phenotypes were recorded within 24 h of admission using a structured clinical assessment and categorized into predefined patterns such as Wind-Phlegm Collateral Obstruction, Phlegm-Heat Fu-Excess, Qi Deficiency and Blood Stasis, Yin Deficiency with Wind Agitation, and others. TCM syndrome differentiation was performed by two attending TCM physicians independently using the standardized “Stroke Diagnosis and Efficacy Evaluation Criteria” (11). Both assessors were blinded to neuroimaging and clinical outcome data. In cases of disagreement, a third senior TCM physician was consulted to reach a consensus.

### 2.3 Outcome measures

The primary outcome was unfavorable neurological status at discharge, defined as NIHSS >8 (12, 13), representing moderate-to-severe neurological impairment. This threshold was selected with reference to prior studies that categorized stroke severity as mild (NIHSS < 8), moderate (NIHSS 8–16), and severe (>16) (14).

Although the modified Rankin Scale at 90 days is widely used to assess long-term functional outcome after stroke, the present study focused on early neurological status during hospitalization. The NIHSS is a validated quantitative measure of stroke-related neurological deficit and is suitable for assessing short-term neurological status in acute ischemic stroke. Using data from the China National Stroke Registry III (CNSR III), Yi et al. (15) supported the utility of NIHSS as an important measure of in-hospital neurological status in acute ischemic stroke. In addition, discharge NIHSS was consistently available in this retrospective cohort, allowing standardized assessment across patients.

### 2.4 Statistical analysis

Continuous variables were analyzed using *t*-tests or Mann–Whitney U-tests, as appropriate. Categorical variables were compared using chi-square tests or Fisher's exact test, as appropriate. Univariate analyses were initially performed to explore associations between baseline variables and unfavorable outcomes. Variables with *p* < 0.05 in univariate analyses were considered as candidate variables for inclusion in the multivariable logistic regression model. Variance inflation factors (VIF) were used to assess multicollinearity among variables, and all VIF values were < 5, indicating no significant collinearity. Statistical significance was set at a two-sided *p* < 0.05. All statistical analyses were performed using SPSS (version 27.0). Data visualization (violin plots) was performed using R software (version 4.4.1). Given the exploratory nature of this study, no adjustment for multiple comparisons was applied, and results should be interpreted conservatively.

## 3 Results

### 3.1 Study population and baseline characteristics

**Figure 1:**
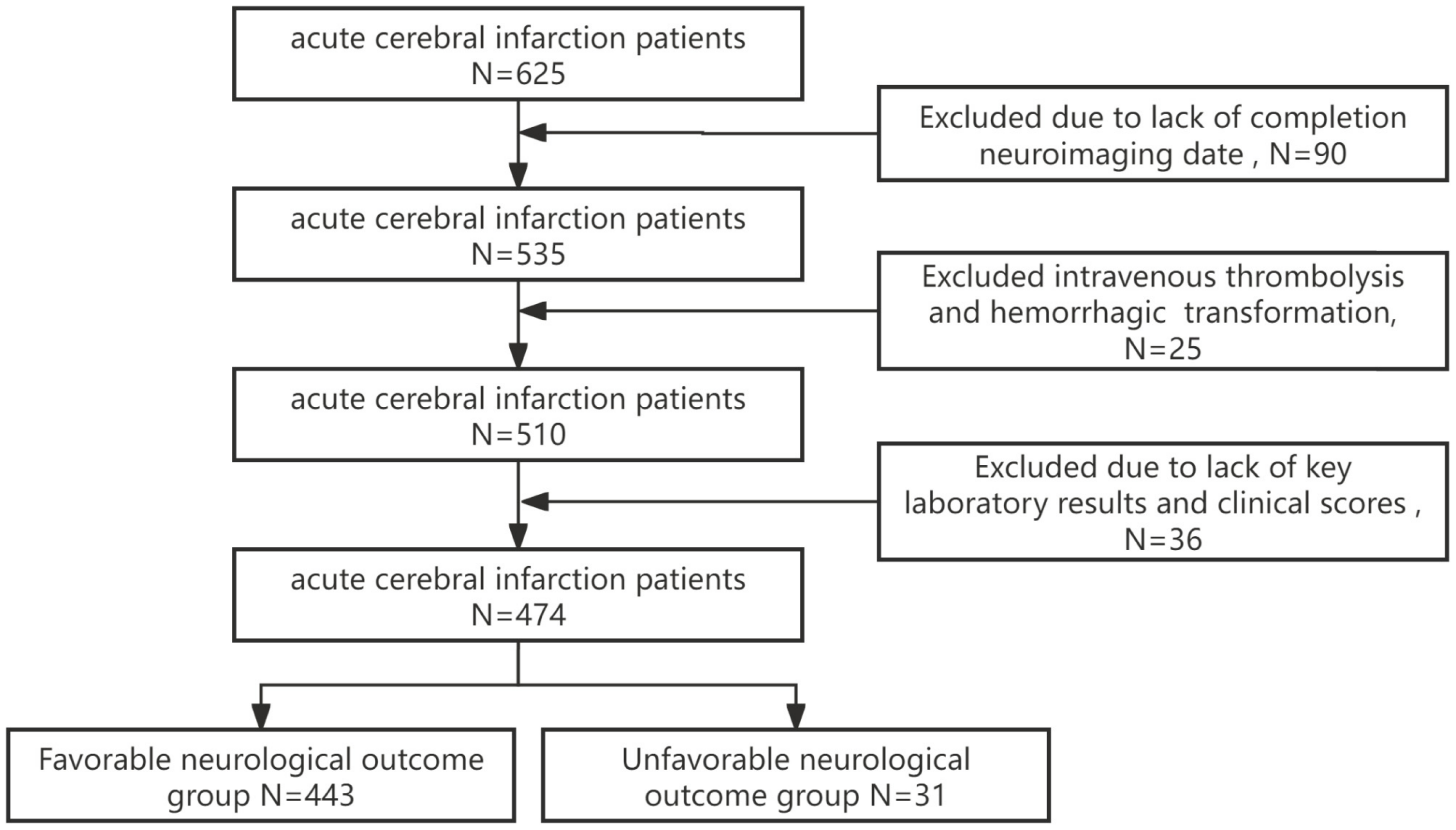
Flowchart of patients included in the study.

A total of 625 patients with AIS were initially screened. After applying inclusion and exclusion criteria, 474 patients were included in the final analysis, 443 had favorable outcomes, and 31 had unfavorable outcomes. The cohort had a median age of 67 years, with 71% being male. Based on neurological functional outcomes at discharge (Figure 1).

There were no significant differences in age, sex, TOAST subtype, or most laboratory parameters (*p* > 0.05). However, significant differences were observed in the prevalence of hyperhomocysteinemia, C-reactive protein (CRP) levels, international normalized ratio (INR), fibrinogen (FIB) levels, as well as in the distribution of the Phlegm-Heat Fu-Excess and Wind-Phlegm Obstructing Collaterals systemic clinical phenotype, and total CSVD burden score (*p* < 0.05). Comparison of baseline characteristics between the two groups is summarized in Table 1.

**Table 1:** Comparison of baseline characteristics between groups with favorable and unfavorable neurological outcomes at discharge.

Characteristic	Favorable outcome (*n* = 443)	Unfavorable outcome (*n* = 31)	Statistics *(Z/*χ^2^)	*p*
Age, years, Median (IQR)	67 (59, 73.5)	67 (61.5, 73)	0.253	0.801
Male, *n* (%)	314 (71)	21 (68)	0.138	0.867
Previous infarction, *n* (%)	146 (33)	14 (45)	1.930	0.233
Hypertension, *n* (%)	344 (78)	27 (87)	1.519	0.314
Diabetes, *n* (%)	216 (49)	16 (52)	0.094	0.903
Coronary artery disease, *n* (%)	112 (25)	9 (29)	0.214	0.803
Atrial fibrillation, *n* (%)	36 (8)	3 (10)	0.000	0.733
Hyperhomocysteinemia, *n* (%)	75 (17)	12 (39)	9.171	0.005
Smoking history, *n* (%)	232 (52)	19 (61)	0.925	0.438
Alcohol use, *n* (%)	185 (42)	12 (39)	0.111	0.885
CRP, mg/L, M (Q1, Q3)	1.23 (0.23, 2.13)	3.17 (0.66, 9.56)	2.445	0.015
Total cholesterol, mmol/L, M (Q1, Q3)	4.23 (3.63, 4.74)	3.66 (3.04, 5.52)	0.955	0.340
Triglycerides, mmol/L, M (Q1, Q3)	1.33 (1.08, 1.76)	1.18 (0.9, 1.73)	1.410	0.159
HDL, mmol/L, M (Q1, Q3)	1.06 (0.94, 1.2)	1.01 (0.86, 1.08)	1.676	0.094
LDL, mmol/L, M (Q1, Q3)	2.63 (2.18, 3.08)	2.63 (1.85, 3.29)	0.276	0.783
Homocysteine, μmol/L, M (Q1, Q3)	12.5 (10.7, 14.9)	12.7 (11.02, 19.84)	1.288	0.198
HBA1C, %, M (Q1, Q3)	6.3 (6.1, 7)	6.3 (5.6, 6.8)	1.007	0.314
PT, seconds, M (Q1, Q3)	12 (11.4, 12.5)	12.1 (11.45, 13.05)	0.754	0.451
INR, M (Q1, Q3)	1.04 (1.01, 1.08)	1.07 (1.02, 1.13)	2.073	0.038
APTT, seconds, M (Q1, Q3)	27.8 (25.5, 29.8)	28.4 (26.3, 30.95)	1.525	0.127
Fibrinogen, g/L, M (Q1, Q3)	3.17 (2.86, 3.53)	3.44 (3.02, 3.99)	2.520	0.012
TT, seconds, M (Q1, Q3)	15.2 (14.7, 15.8)	15.3 (14.6, 16.1)	0.664	0.507
Phlegm-Heat Fu-Excess, *n* (%)	20 (5)	6 (19)	9.611	0.004
Phlegm-stasis obstructing collaterals, *n* (%)	59 (13)	5 (16)	0.029	0.592
Qi deficiency and blood stasis, *n* (%)	41 (9)	4 (13)	0.125	0.521
Wind-Phlegm obstructing collaterals, *n* (%)	159 (36)	5 (16)	5.001	0.041
Yin deficiency with wind agitation, *n* (%)	32 (7)	1 (3)	0.231	0.713
Other syndromes, *n* (%)	132 (30)	10 (32)	2.229	0.215
TOAST classification, *n* (%)			5.518	0.298
Large-artery atherosclerosis	156 (35)	16 (52)		
Small-artery occlusion	225 (51)	11 (35)		
Cardioembolism	40 (9)	4 (13)		
Other determined etiology	9 (2)	0 (0)		
Undetermined etiology	13 (3)	0 (0)		
Total CSVD Burden Score, *n* (%)			11.250	0.016
0	195 (44)	10 (32)		
1	127 (29)	4 (13)		
2	69 (16)	9 (29)		
3	39 (9)	6 (19)		
4	13 (3)	2 (6)		

Variables with *p* < 0.05 in univariate analysis were included in the multivariate logistic regression model. Patients with unfavorable outcomes had higher levels of CRP and other laboratory indicators, greater CSVD burden, and higher prevalence of lacunar infarction. In multivariable analysis, total CSVD burden, hyperhomocysteinemia, and the TCM-defined Phlegm-Heat Fu-Excess phenotype remained associated with an increased likelihood of unfavorable outcomes (Table 2).

**Table 2:** Multivariate logistic regression analysis of factors associated with unfavorable neurological outcome.

Variable	β	SE	Adjust OR	95% *CI*	*p*
History of hyperhomocysteinemia	1.016	0.442	2.763	1.209–6.314	0.016
Total CSVD burden score	0.451	0.166	1.569	1.133–2.174	0.007
Phlegm-Heat Fu-Excess syndrome	1.664	0.534	5.280	1.854–15.039	0.002

### 3.2 Comparison of individual CSVD imaging features

**Figure 2:**
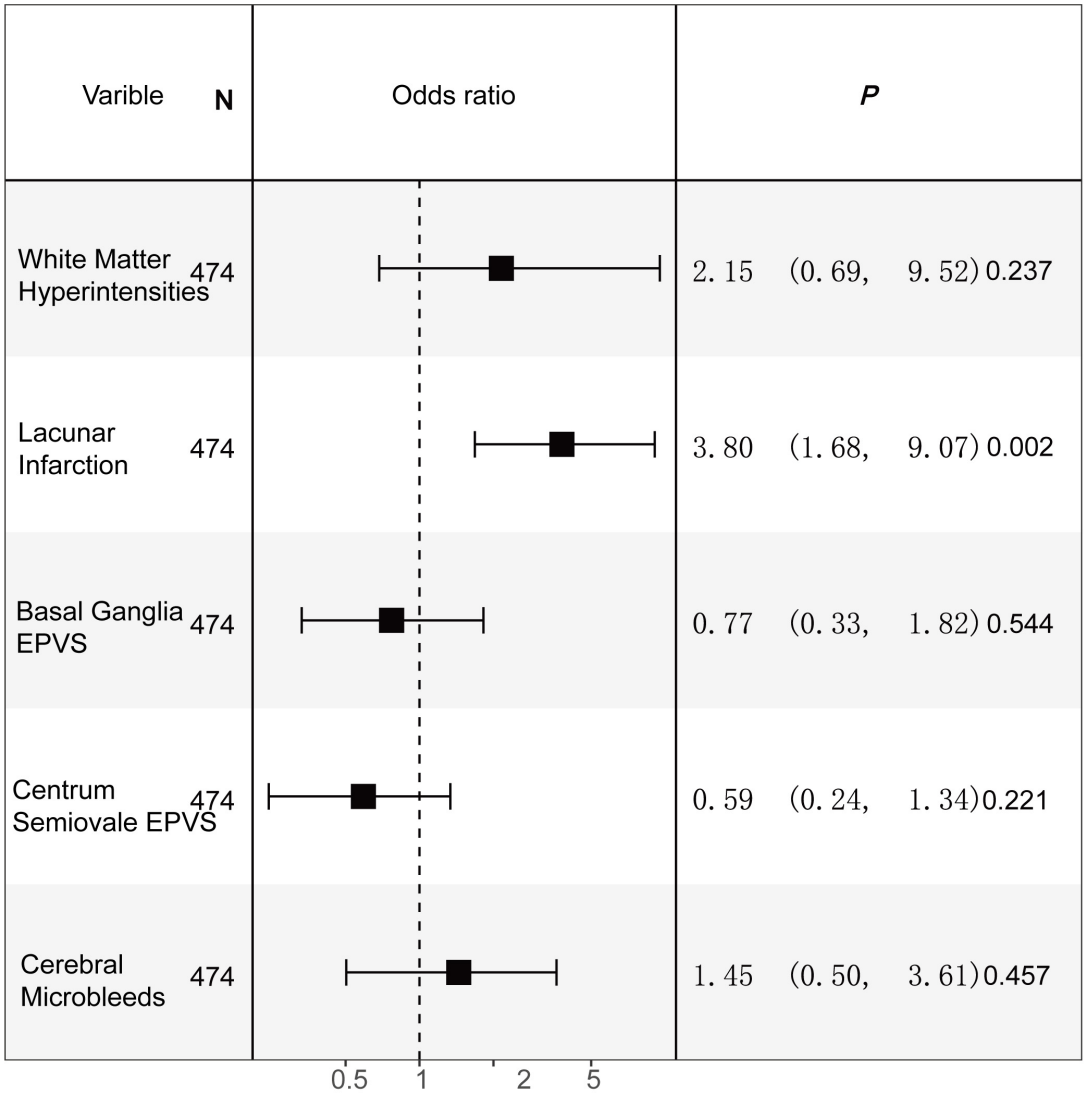
Multivariable logistic regression analysis of cerebral small vessel disease (CSVD) imaging features for unfavorable neurological outcome.

The presence of specific CSVD imaging features was compared between the outcome groups (Table 3). A significantly higher proportion of patients in the unfavorable outcome group had lacunar infarcts (65% vs. 32%, *p* < 0.001). In the multivariate model, lacunar infarction remained an independent predictor of poor outcome (OR = 3.80, 95% CI: 1.68–9.07, *p* = 0.002; Figure 2). The prevalence of moderate-to-severe white matter hyperintensities (90% vs. 75%, *p* = 0.081) and cerebral microbleeds (19% vs. 12%, *p* = 0.261) was higher in the unfavorable outcome group. However, these differences did not reach statistical significance.

**Table 3:** Comparison of CSVD imaging features between outcome groups.

CSVD feature	Favorable outcome group (*n* = 443)	Unfavorable outcome group (*n* = 31)	Statistics *(Z/*χ^2^)	*p*
White matter hyperintensities, *n* (%)	331 (75)	28 (90)	3.037	0.081
Lacunar infarction, *n* (%)	141 (32)	20 (65)	13.802	< 0.001
Basal ganglia EPVS, *n* (%)	231 (52)	18 (58)	0.407	0.651
Centrum semiovale EPVS, *n* (%)	169 (38)	9 (29)	1.027	0.411
Cerebral microbleeds, *n* (%)	54 (12)	6 (19)	0.775	0.261

### 3.3 Association between CSVD features and discharge NIHSS scores

**Figure 3:**
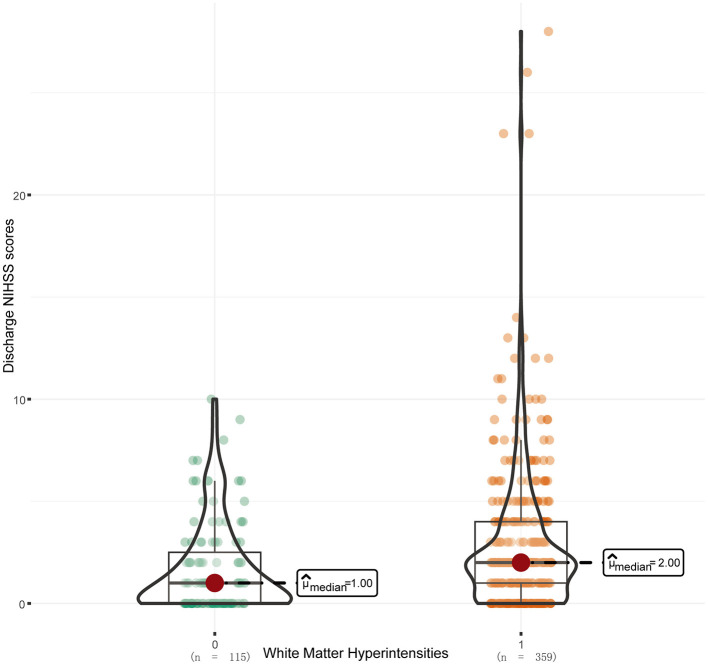
Distribution of discharge NIHSS Scores by presence of white matter hyperintensities. $W_{Mann-Whitney}=15484.50,\;p=3.93\times 10^{-5},\;{\hat{r}}_{biserial}^{rank}=-0.25,\;Cl_{95\%}\left[-0.36,-0.13\right],\;n_{obs}=474\;$.

**Figure 4:**
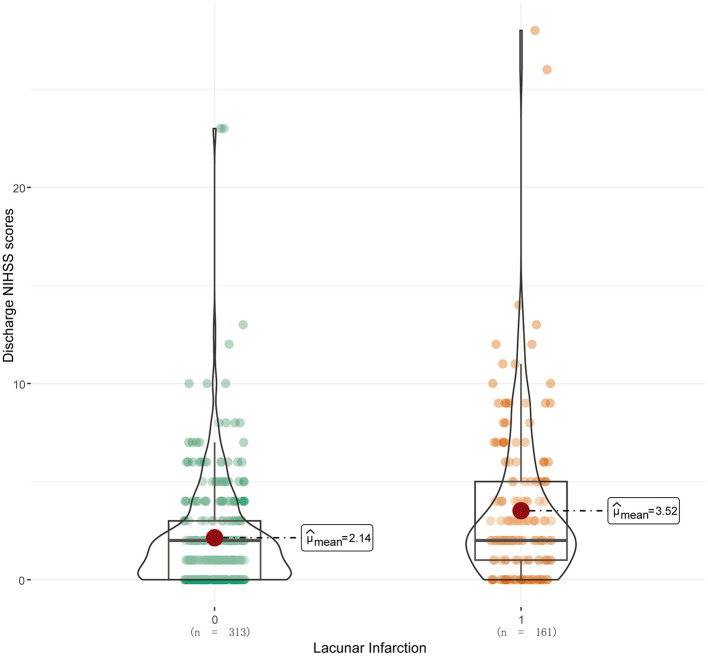
Distribution of discharge NIHSS scores by presence of lacunar infarction. $t_{Welch}\left(239.72\right)=-3.86,\;p=1.46\times 10^{-4},\;{ĝ}_{Hedges}=-0.39,\;Cl_{95\%}\left[-0.60,0.19\right],\;n_{obs}=474\;$ $\log_{e}\left(BF_{01}\right)=-6.72,\;\hat{\delta }\;\begin{array}{c} posterior\\ difference \end{array}=-1.34,\;Cl_{95\%}^{ETI}\left[-1.98,-0.73\right],\;r_{Cauchy}^{JZS}=0.71\;$.

**Figure 5:**
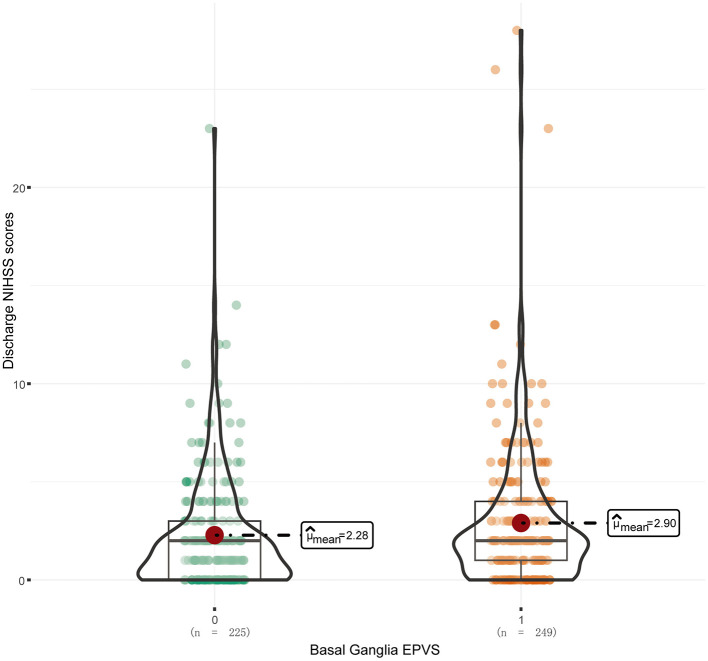
Distribution of discharge NIHSS scores by presence of basal ganglia enlarged perivascular spaces (EPVS). $t_{Welch}\left(466.29\right)=-2.05,\;p=0.040,\;{ĝ}_{Hedges}=-0.19,\;Cl_{95\%}\left[-0.37,30\times 10^{-3}\right],\;n_{obs}=474\;$ $\log_{e}\left(BF_{01}\right)=0.30,\;\hat{\delta }\;\begin{array}{c} posterior\\ difference \end{array}=-0.60,\;Cl_{95\%}^{ETI}\left[-1.17,-0.03\right],\;r_{Cauchy}^{JZS}=0.71\;$.

**Figure 6:**
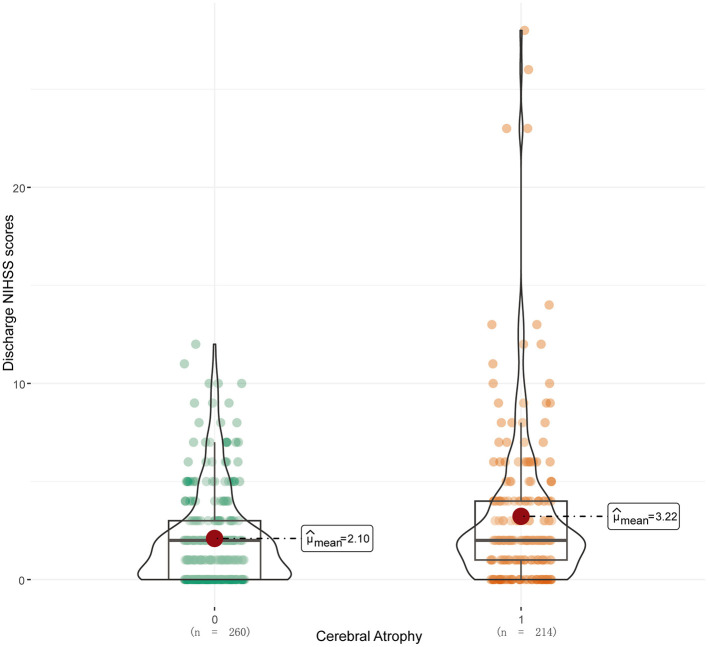
Distribution of discharge NIHSS Scores by presence of cerebral atrophy. $t_{Welch}\left(328.89\right)=-1.06,\;p=4.63\times 10^{-4},\;{ĝ}_{Hedges}=-0.33,\;Cl_{95\%}\left[-0.52,0.15\right],\;n_{obs}=474\;$ $\log_{e}\left(BF_{01}\right)=-4.32,\;\hat{\delta }\;\begin{array}{c} posterior\\ difference \end{array}=-1.09,\;Cl_{95\%}^{ETI}\left[-1.70,-0.49\right],\;r_{Cauchy}^{JZS}=0.71\;$.

**Figure 7:**
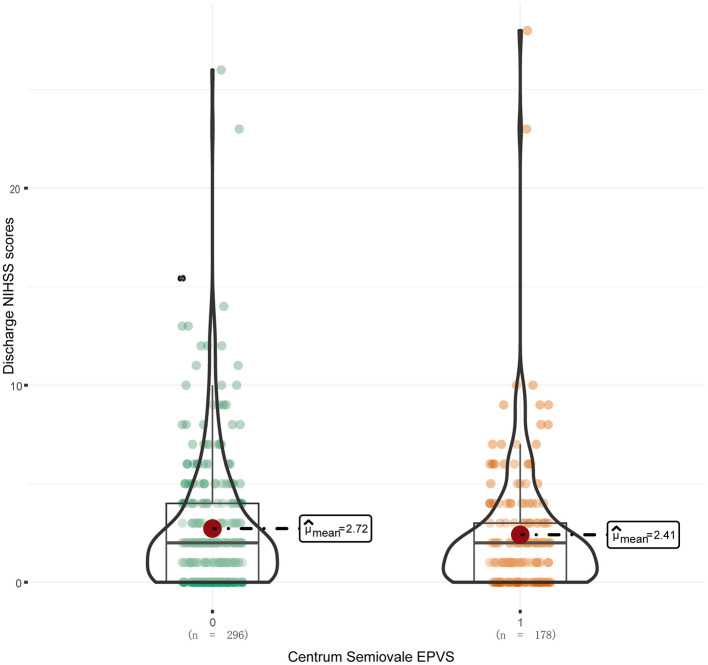
Distribution of discharge NIHSS Scores by presence of centrum semiovale enlarged perivascular spaces. *t*_*Welch*_(370) = 0.99, *p* = 0.330, ĝ_*Hedges*_ = 0.09, *Cl*_95%_[−0.09, 0.28], *n*_*obs*_ = 474 $\log_{e}\left(BF_{01}\right)=1.78,\;\hat{\delta }\;\begin{array}{c} posterior\\ difference \end{array}=0.31,\;Cl_{95\%}^{ETI}\left[-0,34,-0.90\right],\;r_{Cauchy}^{JZS}=0.71\;$.

**Figure 8:**
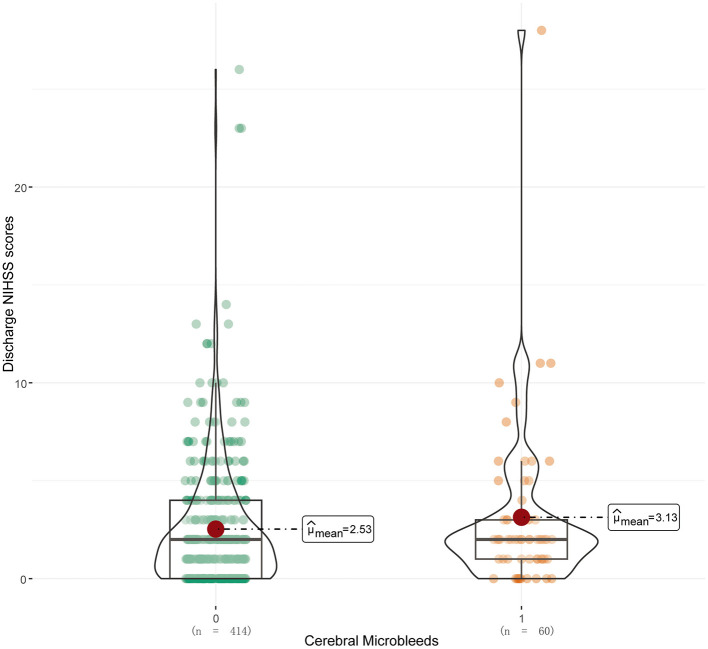
Distribution of discarge NIHSS scores by presence of cerebral microbleeds. *t*_*Welch*_(68.97) = −1.06, *p* = 0.290, ĝ_*Hedges*_ = −0.16, *Cl*_95%_[−0.45, 0.14], *n*_*obs*_ = 474 $\log_{e}\left(BF_{01}\right)=1.09,\;\hat{\delta }\;\begin{array}{c} posterior\\ difference \end{array}=-0.55,\;Cl_{95\%}^{ETI}\left[-1.42,-0.33\right],\;r_{Cauchy}^{JZS}=0.71\;$.

In addition to the primary binary outcome analysis, we performed exploratory analyses examining discharge NIHSS as a continuous variable. Between-group comparisons were performed using the Mann–Whitney U-test, and results are visualized using violin plots (Figures 3–8). These findings should be considered supplementary.

Discharge NIHSS scores were higher in patients with white matter hyperintensities (*p* < 0.001; Figure 3), lacunar infarcts (*p* < 0.001; Figure 4), basal ganglia enlarged perivascular spaces (*p* = 0.040; Figure 5), and cerebral atrophy (*p* < 0.001; Figure 6).

In contrast, no statistically significant difference in NIHSS scores was observed for centrum semiovale enlarged perivascular spaces (*p* = 0.330; Figure 7) or cerebral microbleeds (*p* = 0.290; Figure 8).

## 4 Discussion

This study suggests that higher global CSVD burden and adverse systemic clinical phenotypes are associated with poor short-term neurological outcomes following AIS. These findings support the notion that cumulative microvascular injury may contribute to poorer neurological status during the acute phase.

Among individual CSVD markers, lacunar infarction showed the strongest association with poor outcome, consistent with prior studies suggesting that lacunes may reflect advanced small vessel pathology. White matter hyperintensities and cerebral atrophy were also associated with higher NIHSS scores, possibly reflecting greater structural brain damage.

In the present study, the TCM-defined Phlegm-Heat Fu-Excess phenotype was associated with unfavorable outcomes. Although previous studies have suggested potential links between such clinical patterns and systemic conditions, the underlying mechanisms remain to be clarified. These findings suggest that both neuroimaging markers and systemic clinical characteristics may provide complementary information for early risk assessment. However, these observations should be considered exploratory and require further validation in independent cohorts.

### 4.1 Association between total CSVD burden and neurological outcomes

The association between total CSVD burden and stroke outcomes has been reported in previous studies, particularly in thrombolyzed AIS populations (16). In the present study, higher CSVD burden was associated with unfavorable short-term neurological outcomes, extending these observations to in-hospital recovery. These findings suggest a potential link between pre-existing microvascular injury and short-term neurological outcomes (17).

The CSVD score represents the cumulative effect of chronic cerebral microangiopathy, which could be associated with cerebral resilience and may affect compensatory mechanisms, then could contribute to infarct growth, thereby exacerbating the functional impact of a new infarction (18, 19). This is consistent with the “cumulative damage” hypothesis and suggests that assessment of global CSVD burden provides additional information beyond acute lesion characteristics (20).

### 4.2 Heterogeneous contributions of CSVD imaging features

The impact of distinct CSVD imaging features on neurological outcomes appeared to be heterogeneous. Among the specific CSVD features, lacunar infarcts showed the strongest association with unfavorable outcomes (21, 22). This may be attributed to their location in critical deep brain regions involved in neural networks and white matter tracts, potentially disrupting connectivity and functional integration (23, 24). Furthermore, the presence of lacunes may signify a more advanced and widespread small vessel pathology, leading to impaired cerebrovascular reactivity and compromised collateral flow during the acute ischemic period (25).

Previous studies have established that a higher burden of white matter hyperintensities (WMH) is associated with increased stroke severity and influences functional recovery across multiple domains after acute ischemic stroke (26, 27). Consistent with these reports, our study found that patients with moderate-to-severe WMH exhibited higher median NIHSS scores at discharge. Concomitant WMH-related injury may impair the capacity for neurological recovery following corticospinal tract damage, aligning with the “white matter integrity decline–cognitive-motor impairment” theory proposed by Wang et al. (28). WMH likely disrupts long-range white matter tracts—such as the corpus callosum and corticospinal tract—thereby reducing neural conduction efficiency (29). In our cohort, nearly all patients with NIHSS scores >10 had observable WMH, suggesting that WMH may serve as a potential marker for severe neurological impairment.

### 4.3 Biological interpretation of Phlegm-Heat Fu-Excess syndrome from an integrated Chinese-Western medical perspective

The significant association between Phlegm-Heat Fu-Excess syndrome and unfavorable outcome provides an additional clinical perspective for outcome assessment (30). This syndrome, characterized by systemic manifestations of heat, phlegm, and bowel excess, has been hypothesized in the TCM literature to be associated with a state of heightened inflammation, oxidative stress, and dysautonomia—processes known to exacerbate ischemic injury and impede recovery (31, 32). However, direct measurements of these biological pathways were not available in this study; therefore, the interpretation remains hypothesis-generating rather than confirmatory. The observed effect size (OR > 5) should be interpreted with caution given the limited number of outcome events, and further studies are needed to validate its clinical relevance and explore potential underlying mechanisms.

### 4.4 Hyperhomocysteinemia is a shared risk factor for both macrovascular and microvascular disease

In our study, hyperhomocysteinemia was associated with unfavorable neurological outcomes (OR = 2.76, 95% CI: 1.21–6.31). This finding is consistent with previous reports linking elevated homocysteine levels to CSVD burden and worse stroke outcomes. Hyperhomocysteinemia has been reported as a risk factor for various cardio-cerebrovascular diseases and has been associated with both macrovascular and microvascular pathology (33). In the context of CSVD, hyperhomocysteinemia impairs endothelial function at early stages via oxidative stress, inflammatory pathways, and epigenetic modifications—often preceding overt microvascular injury and clinical manifestation (34). Elevated homocysteine levels have been associated with lacunar infarcts and a higher burden of white matter hyperintensities in CSVD (35). However, given the observational nature of our study, these results should be interpreted as associative rather than causal, and further studies are required to clarify the underlying mechanisms.

### 4.5 Limitations and future directions

Several limitations of this study should be acknowledged. First, its retrospective design introduces the potential for selection and information bias. The exclusion of patients receiving reperfusion therapy may introduce selection bias and limit the generalizability of our findings. Second, the relatively small number of patients in the unfavorable outcome group may have limited the statistical power to detect significant associations for less prevalent imaging features, such as microbleeds. At the same time, given the limited number of outcome events, only a small number of variables were included in the final multivariable model, and the stability of the model may be limited. Third, the use of a dichotomized outcome based on discharge NIHSS, while clinically relevant, does not capture long-term functional recovery. Baseline stroke severity (e.g., admission NIHSS) was not included in the final multivariable model due to its strong correlation with the outcome variable (discharge NIHSS), which may result in over-adjustment. However, residual confounding cannot be excluded. Fourth, systemic clinical phenotypes, though based on standardized criteria, possess an inherent element of subjectivity. Finally, CSVD markers were assessed using visual rating, which may introduce subjectivity. Inter-rater reliability for CSVD marker assessment was not formally calculated. Although standardized criteria were applied, inter-rater variability cannot be fully excluded. Future studies incorporating quantitative imaging methods and formal reproducibility assessment are warranted.

Future studies should prioritize prospective, multicenter designs with larger sample sizes to validate our findings. Incorporating long-term functional outcomes and objective biomarker assessments would strengthen the mechanistic understanding of the observed associations. Moreover, exploring the interplay between systemic clinical phenotypes and quantitative neuroimaging biomarkers could pave the way for developing integrated prognostic models. Finally, interventional studies examining whether early TCM-based interventions, such as herbal formulas targeting Phlegm-Heat Fu-Excess, may improve outcomes in high-risk patients are warranted.

## 5 Conclusions

Global CSVD burden and systemic clinical phenotypes were associated with unfavorable short-term neurological outcomes after AIS. Integrating neuroimaging markers and systemic clinical characteristics may provide additional information for early risk assessment, but requires further validation in larger prospective studies.

## Data Availability

The raw data supporting the conclusions of this article will be made available by the authors, without undue reservation.
